# European Society for Immunodeficiencies (ESID) and European Reference Network on Rare Primary Immunodeficiency, Autoinflammatory and Autoimmune Diseases (ERN RITA) Complement Guideline: Deficiencies, Diagnosis, and Management

**DOI:** 10.1007/s10875-020-00754-1

**Published:** 2020-02-17

**Authors:** Nicholas Brodszki, Ashley Frazer-Abel, Anete S. Grumach, Michael Kirschfink, Jiri Litzman, Elena Perez, Mikko R. J. Seppänen, Kathleen E. Sullivan, Stephen Jolles

**Affiliations:** 1 Department of Pediatrics, Children’s Hospital, Skåne University Hospital, Lund, Sweden; 2 Division of Rheumatology, Department of Medicine, University of Colorado School of Medicine, Aurora, CO USA; 3 Clinical Immunology, Reference Center on Rare Diseases, University Center Health ABC, Santo Andre, SP Brazil; 4 Institute of Immunology, University of Heidelberg, Heidelberg, Germany; 5 Department of Clinical Immunology and Allergology, St Anne’s University Hospital, and Faculty of Medicine, Masaryk University, Brno, Czech Republic; 6 Allergy Associates of the Palm Beaches, North Palm Beach, FL USA; 7 Rare Disease Center, Children’s Hospital, and Adult Primary Immunodeficiency Outpatient Clinic, Inflammation Center, University of Helsinki and Helsinki University Hospital, Helsinki, Finland; 8 Division of Allergy and Immunology, The Children’s Hospital of Philadelphia, Philadelphia, PA USA; 9 Immunodeficiency Centre for Wales, Cardiff University & University Hospital of Wales, Cardiff, UK

**Keywords:** Complement, complement deficiencies, classical pathway, alternative pathway, mannan-binding lectin

## Abstract

This guideline aims to describe the complement system and the functions of the constituent pathways, with particular focus on primary immunodeficiencies (PIDs) and their diagnosis and management. The complement system is a crucial part of the innate immune system, with multiple membrane-bound and soluble components. There are three distinct enzymatic cascade pathways within the complement system, the classical, alternative and lectin pathways, which converge with the cleavage of central C3. Complement deficiencies account for ~5% of PIDs. The clinical consequences of inherited defects in the complement system are protean and include increased susceptibility to infection, autoimmune diseases (e.g., systemic lupus erythematosus), age-related macular degeneration, renal disorders (e.g., atypical hemolytic uremic syndrome) and angioedema. Modern complement analysis allows an in-depth insight into the functional and molecular basis of nearly all complement deficiencies. However, therapeutic options remain relatively limited for the majority of complement deficiencies with the exception of hereditary angioedema and inhibition of an overactivated complement system in regulation defects. Current management strategies for complement disorders associated with infection include education, family testing, vaccinations, antibiotics and emergency planning.

## Introduction

Most complement deficiencies have a combined estimated prevalence of 0.03% in the general population, meaning that they meet the criteria for rare diseases (< 0.05% in the EU and < 200,000 individuals in the USA [i.e., approximately < 0.06%]) [1]. A small number of deficiencies are more common: mannose-binding lectin (MBL) deficiency has a prevalence of ~5%, and deficiencies of C4A and C4B have prevalence rates of 11–22% and 30–45%, respectively [2, 3]. Complement deficiencies collectively account for 5.2% of the primary immunodeficiencies (PIDs) reported in the European Society for Immunodeficiencies (ESID) Registry and may well be underestimated partly due to a lack of readily available laboratory testing [4, 5].

The complement system is a highly conserved part of the innate immune system, which can be traced back as far as the sea urchin [6]. It comprises over 30 membrane-bound and soluble components and has three major functions: (1) host defense by opsonisation, chemotaxis, induction of inflammation and lysis of targets [7–10] (2) interfacing between innate and adaptive immunity by augmenting the antibody response and immunological memory [11, 12] and (3) the disposal of waste through the clearance of apoptotic cells and immune complexes [13–17].

**Fig. 1:**
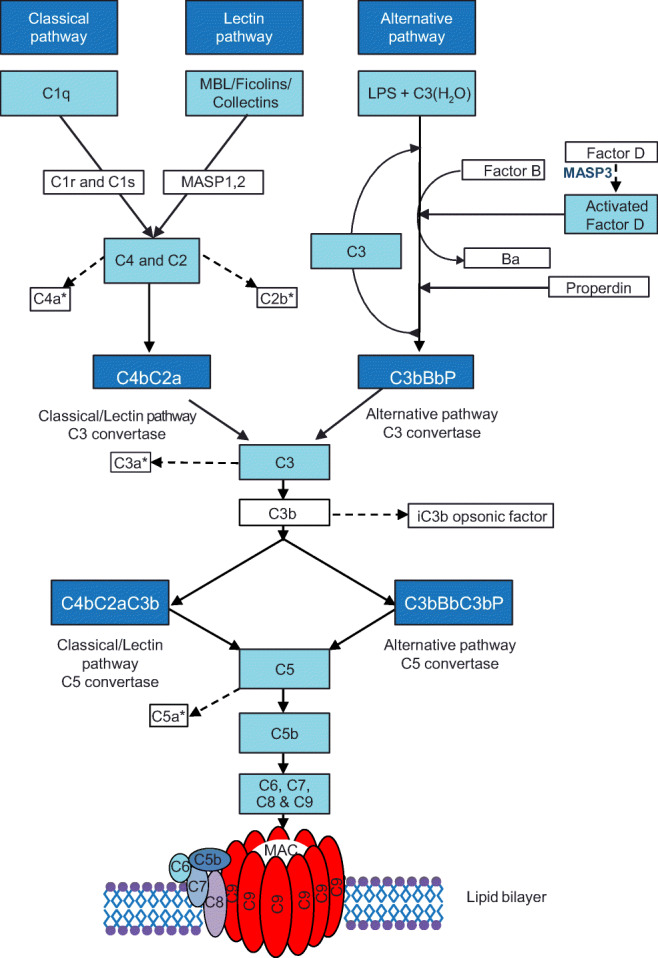
Complement system activation pathways. LPS lipopolysaccharide, MAC membrane attack complex, MASP mannose-binding lectin-associated serine protease, MBL mannose-binding lectin. *Examples of anaphylatoxins from the complement pathways

The complement system is organized into three distinct enzymatic cascade pathways, namely the classical, alternative and lectin pathways [7, 10, 13, 15, 18–20] (Fig. 1). Each of these converge toward the cleavage of central C3 by a C3 convertase, followed by the formation of a C5 convertase, which cleaves C5 into C5a and C5b [17, 21]. This results in activation of the common lytic effector terminal pathway [14]. The subsequent insertion of terminal pathway components into the cell wall leads to lysis via the membrane attack complex (MAC), which is composed of complement proteins C5b–9 [15, 17, 21, 22]. This can occur in both bacterial and human cells, e.g., cancer cells, and, in turn, leads to the release of biologically active fragments that enhance inflammation, recruit leukocytes and promote host defense [13, 23]. These proinflammatory cascades require strict control by a range of soluble and membrane-bound regulatory proteins that act to limit complement-mediated damage to the host [10, 15, 24, 25].

The clinical consequences of inherited defects in the complement system fall broadly into three areas: (1) increased susceptibility to infection caused by encapsulated organisms, (2) autoimmunity, in particular systemic lupus erythematosus (SLE) [26] and (3) hyperactivation due to deficiencies in regulatory proteins that result in specific disorders.

This guideline aims to describe the complement system, the functions of the constituent pathways, regulatory proteins and the expanding range of disorders associated with complement defects, with particular focus on PIDs and their diagnosis and management. It represents consensus opinions of the authors, based on the current literature and available evidence.

The classical pathway is triggered by activation of the subunits of C1 (q, r and s) after binding of C1q to immunoglobulin-G (IgG) and IgM-containing immune complexes or by C-reactive protein (CRP). The lectin pathway is activated by the interaction of a protein molecule (MBL, collectin-10, collectin-11, ficolin-1, ficolin-2 or ficolin-3) with carbohydrate residues on bacterial surfaces. This results in the activation of MBL-associated serine protease 1 (MASP1) and MASP2. The alternative pathway is continuously undergoing low grade spontaneous activation “tick over” through a feedback loop involving hydrolysis of C3 and may also be triggered by LPS derived from Gram negative bacteria. The membrane attack complex (MAC) is formed by the sequential assembly of C5b, C6, C7, C8 and many copies of C9 into a pore.

## Assessment of the Complement System

The first tier of complement system assessment is to carry out functional assays that are specific for each activation arm. By performing assays relating to the classical, alternative and lectin pathways, it is possible to screen for and narrow down the possible deficiencies [18, 27]. Following detection of absent or low activity in one or more pathways, the search can be focused on specific deficiencies [28]. Accordingly, the next tier of testing is to measure the concentration and/or function of individual components [1]. During testing, it is important to note that complement levels reflect a balance between consumption through activation and production because many are acute phase reactants [29]. It is exceedingly rare to have more than one component deficient in the same individual, with the exception of those of the lectin pathway (MBL, mannose-binding lectin-associated serine protease 2; MASP2). Therefore, if multiple components are low, it is possible that sample handling was improper, a regulatory protein was deficient or autoantibodies were present. Autoantibodies targeting the complement system may need to be assessed because they are often associated with specific diseases, e.g., anti-C1q antibody is associated with hypocomplementemic urticarial vasculitis and/or proliferative SLE nephritis [26, 30].

**Fig. 2:**
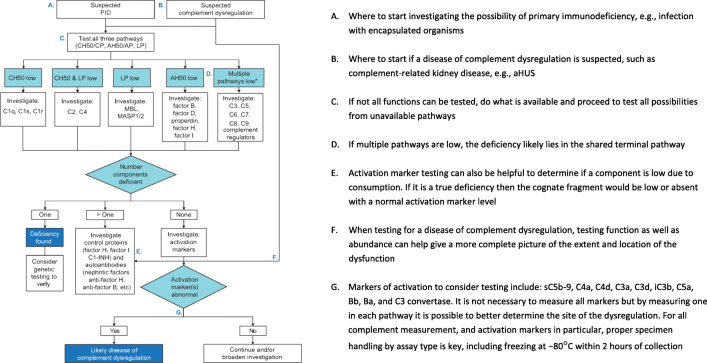
Algorithm for complement testing. **a** Where to start investigating the possibility of primary immunodeficiency, e.g., infection with encapsulated organisms. **b** Where to start if a disease of complement dysregulation is suspected, such as complement-related kidney disease, e.g., aHUS. **c** If not all functions can be tested, do what is available and proceed to test all possibilities from unavailable pathways. **d** If multiple pathways are low, the deficiency likely lies in the shared terminal pathway. **e** Activation marker testing can also be helpful to determine if a component is low due to consumption. If it is a true deficiency then the cognate fragment would be low or absent with a normal activation marker level. **f** When testing for a disease of complement dysregulation, testing function as well as abundance can help give a more complete picture of the extent and location of the dysfunction. **g** Markers of activation to consider testing include: sC5b-9, C4a, C4d, C3a, C3d, iC3b, C5a, Bb, Ba and C3 convertase. It is not necessary to measure all markers but by measuring one in each pathway it is possible to better determine the site of the dysregulation. For all complement measurement, and activation markers in particular, proper specimen handling by assay type is key, including freezing at − 80°C within 2 h of collection. AH50, alternative pathway hemolytic activity; aHUS, atypical hemolytic uremic syndrome; AP, alternative pathway; C1-INH, C1 esterase inhibitor; CH50, complement hemolytic activity; CP, classical pathway; LP, lectin pathway; MASP, MBL-associated serine protease; MBL, mannose-binding lectin; PID, primary immunodeficiency. *The sample may have been improperly handled, or the patient has autoantibodies against complement components.

The measurement of complement activation fragments may enable complement consumption (due to overactivation) to be distinguished from (partial) deficiency and show subtle changes in response to complement dysregulation [30]. These activation markers indicate which pathways are involved and to what extent, as well as the degree of therapeutic complement inhibition. Activation markers include fragments of components that are formed during cleavage, e.g., C3a, Bb, complexes of the activated component and their respective regulators, e.g., sC5b–9, and convertases that are formed by activation [30]. The measurement of these markers is complicated by short half-lives and in vitro activation; the cascade will activate in the tube after blood draw if not promptly frozen at − 80 °C [31]. Samples should therefore be frozen within 2 h. This makes pre-analytical specimen handling critical for complement analysis. Ethylenediaminetetraacetic acid (EDTA) can largely inhibit in vitro activation; however, it is important to verify tube type by assay since samples preserved in EDTA are not usually compatible with complement functional testing. Fig. 2 shows a flow diagram outlining the steps that should be undertaken during complement testing.

There is huge variation between laboratories with regard to complement testing, and few standardized tests are available (with the exception of C3 and C4 levels). Given the importance of complement proteins in a wide range of biological systems, effective, standardized and accessible tests are needed [28].

Nephelometric or immunoprecipitation assays are commonly used to measure concentrations of individual components of the complement system. Hemolytic assays for testing the functions of the classical pathway (CH50) and the alternative pathway (AH50) have been available for over 40 years and are based on quantifying the amount of serum needed to lyse 50% of a sample of erythrocytes [32]. Similar methodology was also developed for assessment of the lectin pathway [33]. A Slovenian study showed that patients with C2 or C8 deficiencies present with CH50 values below the lower limit of the reference range, that homozygous mutations are associated with lower CH50 values than heterozygous mutations and that patients with infections have lower CH50 values than those not exhibiting infections [34]. Patients with homozygous C2 mutations consistently present with CH50 below the lower limit of the reference range and also significantly lower CH50 than carriers of heterozygous mutations causing C2 deficiency [34]. Hemolytic assays are time-consuming and have the potential to produce variable results. Methods based on enzyme-linked immunoassay (ELISA) have therefore been developed for all three complement pathways (i.e., the lectin pathway as well as the classical pathway and the alternative pathway) [32, 35, 36]. A single assessment using three enzyme immunoassays to test all three pathways is now commercially available. Activation marker testing may be based either on the detection of zymogen molecules and their products after separation by size (polyclonal antibodies are used in this strategy) or detection of amino acid sequences that are exposed only when the zymogen molecule is activated (neoepitope-specific monoclonal antibodies are used for this approach) [32]. In addition, ELISA methodology has been harnessed to enable assessment of autoantibodies to individual complement components. However, there is huge variation between laboratories regarding preferred methods for complement testing, and few standardized methods have been implemented widely (with the exception of tests for C3 and C4 levels). Given the importance of complement proteins in a wide range of biological systems, effective, standardized and accessible tests are needed [28].

Due to close associations between certain genetic variants and complement deficiencies, genetic testing may be included in the diagnostic work-up [1]. In particular, genetic testing can be used for confirmation when a single component deficiency is identified from quantitative and functional tests (Fig. 2). In recent years, diagnosis by genetic testing has become more common [34], and it offers an alternative option where detailed functional assays are not available or not easily accessible. However, the genetic diagnosis of complement deficiencies is complicated and potentially confounded by copy number variations, point mutations and the presence of pseudogenes [37].

Sanger sequencing allows selective incorporation of chain-terminating dideoxynucleotides during in vitro DNA replication and is widely used to detect single nucleotide variants. Although Sanger sequencing is a valuable technique, relatively high cost and low speed restrict its application in genetic testing [38]. Next-generation sequencing (NGS) is advantageous because it offers high-throughput, rapid and accurate testing of multiple genes simultaneously [39]. The implementation of NGS into routine practice is challenging due to cost to healthcare payers (though costs are steadily reducing over time) and the difficulties in assigning pathogenicity to the novel variants, which are identified [40]. Clincal-exome and whole-exome sequencing are potentially useful methods for diagnosing complement deficiencies as these permit the use of virtual gene panels to detect clinically relevant variants and the interpretation of the results of such panels can be updated alongside functional data to inform the classification of novel potentially pathogenic variants as they are discovered. Over time, whole-genome sequencing will become more accessible, and it is likely that NGS will form an increasing part of complement diagnostics as costs come down and access to testing alongside appropriate bioinformatic and clinical interpretative support becomes more widespread [41].

### Overview of Complement Deficiencies and Diseases

Complement deficiencies make up 1–10% of all reported PIDs, according to international registry data [1]; however, in some national registries, the proportion is significantly higher [34]. Table 1 outlines known complement deficiencies and associated symptoms/disorders. Many of the deficiencies are associated with increased susceptibility to infection (e.g., by encapsulated bacteria) [26]. Deficiency of early components of the classical pathway (C1q, C1r, C1s, C2 and C4) leads to autoimmunity, mainly [42]. Deficiency of C3 and its regulators (factor H [FH] and I [FI]) has been associated with severe recurrent bacterial infections and autoimmunity [43]. Genetic variants in the CFH and CFI genes that lead to haploinsufficiency (i.e., ~50% reductions in the levels of FH and FI) have been associated with age-related macular degeneration (AMD) [44, 45]. These variants underpin the complexity and range of clinical manifestations, relating to both the degree of impairment and the involvement of multiple complement pathways in the pathogenesis of AMD [1, 46, 47]. Properdin and terminal component deficiencies result in an increased risk of neisserial infections [48]. C1-INH deficiency is not thought to be associated with increased susceptibility to infection; however, hereditary angioedema (HAE) attacks can be triggered by the presence of infection [49].

**Table 1:** Complement deficiencies

Deficiency	Gene	Inheritance	Published number of patients	Associated symptoms/disorders
C1q	1p36	AR	~70 patients	SLE, systemic infections with encapsulated organisms; Heterozygous C2 deficiency may have a reduced CH50 but remain asymptomatic
C1r/s (often combined)	12p13	AR	~10 patients	
C2	6p21	AR	1:20,000	
C4 (total C4 deficiency)	6p21	AR	~30 patients	SLE, RA, systemic infections with encapsulated organisms
C4A or C4B	MHC class III region on the short arm of chromosome 6	Complex	1:250	Susceptibility to lymphoma, sarcoidosis, SLE, coeliac disease; prolonged post-infectious symptoms; intolerance to sulphonomides and doxycycline
C3 GoF	19p13	AD	2–8% of aHUS patients	aHUS
C3	19p13	AR	~40 patients	Pyogenic infections, neisserial infections, glomerulonephritis, AMD
Factor H	1q32	AR	< 30 patients	
Factor I	4q25	AR	Rare	
C5	9q33–34	AR	Rare	Neisserial infections; recurrent meningitis
C6	5p13	AR	~1:2000 Afro-Americans. Rare in Caucasians	
C7	5p13	AR	~1:10,000 in Morrocan Jews. Rare in other populations	
C8α–γ/C8β	C8α/β: 1p32 C8γ: 9q34	AR	Rare	
C9	5p14–p12	AR	1:1000 in Japan	Neisserial infections (mostly asymptomatic)
Factor B	6p21	AR	One case	Neisserial and pneumococcal infections, aHUS
Factor D	19p13	AR	2 families	Bacterial infections
MBL	10q11	Polymorphism	5%	Data regarding clinical impact of MBL deficiency are contradictory. Possible effects include susceptibility to bacterial infections and to autoimmunity
Ficolin 3 (H-ficolin)	1p36	Polymorphism	< 10 patients	Various clinical phenotypes
MASP1	3q27	AR	Rare	3MC syndrome
MASP2	1p36		0.03%	Respiratory infections, mostly asymptomatic
C1 inhibitor	11q11–q13	AD	1:50,000	HAE with C1-INH deficiency
C4-binding protein	1q32	Unknown	One case	Atypical Morbus Behçet, angioedema, protein S deficit
Properdin	Xp11	X-linked recessive	Rare	Meningitis (*Neisseria*)
CFHR1–3 deletion	1q32	Complex	Variable (UK 3.4%)	aHUS*, SLE and protection from AMD and IgA nephropathy
Thrombomodulin (CD141)	20p11	AD	Rare	aHUS
CD46/MCP	1q32	Most often heterozygous or compound heterozygous mutations	Rare	aHUS
CD55/DAF	1q32	AR	1–2 cases per million	**PNH
CD55/DAF	1q32	AR	Rare	Protein losing enteropathy CHAPEL syndrome
CD59	11p13	AR	1–2 cases per million	**PNH
CD59	11p13	AR	< 20 patients	Chronic hemolysis and relapsing peripheral demyelinating disease, cerebral infarction
CR2 (CD21)	1q32	AR	Rare	Infections, associated with CVID
CR3 (CD18/CD11b) CR4 (CD18/CD11c, LFA-1)	CD18: 21q22 CD11b: 16p11 CD11c: 16p11	AR	1:1 million	LAD

Table adapted from Grumach & Kirschfink [1]; additional data were obtained from Rosain et al. [50]; Skattum et al. [51]; Degn et al. [13]; Pettigrew et al. [52]; Al-Herz et al. [29]; Zhang et al. [53]; Hamilton et al. [54]; Shiang et al. [55]; Aygören-Pürsün et al. [56]; Liesmaa et al. [57]; National Hemophilia foundation [58] and Nakar et al. [59]; Bork et al. [60]; Holmes et al. [61]

In cases where the prevalence is listed as rare, no numerical value could be idenfitied

*Often associated with anti-factor H antibody (ab) and deficiency of complement factor H-related proteins and autoantibody positive (DEAP) and/or FH-positive hemolytic uremic syndrome

**PNH may be caused by somatic mutation of the Phosphatidylinositol N-acetylglucosaminyltransferase subunit A (PIG-A) gene coding for the enzyme N-acetylglucosaminyltransferase, which is needed for the formation of the glycosylphosphatidylinositol (GPI) anchor of various membrane molecules, such as CD55 and CD59 [62]

*aHUS* atypical hemolytic uremic syndrome, *AMD* age-related macular degeneration, *AD* autosomal dominant, *AR* autosomal recessive, *C1-INH* C1 esterase inhibitor, *C3G* C3 glomerulopathy, *CVID* common variable immunodeficiency, *DAF* decay-accelerating factor, *CFHR* complement factor H-related protein, *GoF* gain of function, *HAE* hereditary angioedema, *LAD* leukocyte adhesion deficiency, *LFA* lymphocyte function-associated antigen 1, *MASP* mannose-associated serine protease, *MBL* mannose-binding lectin, *MCP* membrane cofactor protein, *PNH* paroxysmal nocturnal hemoglobinuria, *RA* rheumatoid arthritis, *SLE* systemic lupus erythematosus

## Classical Pathway

### C1, C2 and C4 Deficiency

The first protein in the classical pathway is C1, which comprises one C1q molecule, two C1r molecules and two C1s molecules [10, 19, 63]. C1q binds to the Fc region of IgM and IgG antibodies and other molecules like CRP bound to target antigens, e.g., viruses, bacteria or autoantigens. The binding of more than one C1q head activates C1r, which then cleaves and activates C1s [21]. Activated C1s cleaves C4 and C2, leading to the formation of C3 convertase [15]**.** This causes large-scale cleavage of C3, and, consequently, the surface becomes coated with C3b molecules, while C3a molecules initiate a localized inflammatory response. IgG subclasses 1 and 3 fix complement more efficiently than IgG2, while IgG4 has no activity in relation to the complement pathway. Patients deficient in the initial components of the classical pathway are prone to autoimmune connective tissue diseases, such as SLE, and other autoimmune diseases, e.g., dermatomyositis, Henoch-Schönlein purpura, juvenile rheumatoid arthritis and glomerulonephritis [64].

Autoimmune manifestations are frequently encountered in patients with C1q deficiency with approximately 55% fulfilling the criteria for SLE, a further 22.5% for SLE-like syndrome and only 7% without evidence of autoimmunity [65]. In individuals with C1r/C1s deficiency, autoimmune manifestations occur in 60–66% and in 75% of patients with complete C4 deficiency [26]. The lowest frequency of autoimmunity (10–42%) is observed in C2-deficient patients [26, 66]. Patients with C1, C2 or C4 deficiency have an increased occurrence of autoantibodies; antinuclear antibodies are present in 75% of patients with C1 or C4 deficiency and 25–55% of patients with C2 deficiency. Anti-dsDNA antibodies are present in 20% of patients with C1q/C4 deficiency and 33% of patients with C2 deficiency [26]. There is a high frequency of the C2 null allele in the Caucasian population (1%) [42]; however, individuals with heterozygous C2 or C4 deficiency often remain asymptomatic [66]. Among individuals with C2 deficiency, the risk of SLE has been reported to be higher in females than males; the female:male ratio of 7:1 is comparable with that seen in the overall population of SLE patients (9:1) [26, 67].

There is an increased incidence of infections in patients with defects of C1, C2 and C4. It is estimated that ~50% of patients develop severe bacterial infections including meningitis, pneumonia, osteomyelitis or septicemia. These infections are caused by encapsulated bacteria, most commonly *Streptococcus pneumoniae*. Significant infections were described in 29/71 (41%) patients with C1q deficiency [65] and in 75% of patients with C2 deficiency [66]. These patients, regardless of the phenotype (autoimmunity or infection) leading to diagnosis, often experience infection and vascular disease as the leading cause of death [66].

### C3 Deficiency

Patients with C3 deficiency are prone to severe infectious complications, e.g., pneumonia, meningitis, osteomyelitis or bacteremia caused by encapsulated bacteria, e.g., *Haemophilus influenzae* and *Neisseria meningitidis*. These infections develop early in life and have a tendency to recur [43, 68]. Patients may also experience membranous glomerulonephritis, while symptoms consistent with SLE are less frequent [69]. Rare C3 gain of function (GoF) mutations may lead to atypical hemolytic uremic syndrome (aHUS) [70]. One common and several rare variants in C3 have been associated with increased risk of AMD [46].

### Deficiency of Terminal Components

Terminal components are shared by the classical, lectin and alternative pathways and are ultimately responsible for the formation of the MAC [71]. The risk of developing meningococcal meningitis is markedly higher in people who have a deficiency of one terminal component compared with the general population, ranging from 1400 times in patients with C9 deficiency to 7000–10,000 times in those with other terminal component deficiencies [43]. In contrast to the immunocompetent population (median age for meningococcal infection: 3 years), the onset of symptoms in patients with terminal deficiencies is 17 years. However, infections generally lead to lower mortality and have a milder course than in immunocompetent persons [42, 43]. Disseminated *Neisseria gonorrhoeae* infections have also been described [42]; however, increased frequency of other bacterial infections is not observed. A terminal component deficiency is more likely if there is a family history of meningococcal infections or repeated neisserial infections or if the causative meningococcal serotype is W-135, X, Y or Z, which less frequently cause infections in healthy individuals.

## Alternative Pathway

The alternative pathway is a highly conserved surveillance system that is continuously turning over (tick-over) due to a labile thioester bond in C3 and thus does not require antibodies or lectins for activation [21]. Properdin is a positive regulator of alternative pathway activity and works by stabilizing alternative pathway convertases [11, 72]. Properdin deficiency is a rare, hereditary, primary immunodeficiency (total number of known cases globally > 100) and is the only X-linked complement deficiency [72]. These patients are unusually susceptible to *Neisseria* infections [72, 73]. It manifests with either complete absence of the molecule (type I), partial deficiency (type II) or a normal level of dysfunctional protein (type III). Properdin-deficient individuals are susceptible to meningococcal disease, which is frequently complicated by sepsis and most commonly occurs in adolescence [48]. The risk of meningococcal infection in healthy individuals is usually greatest in children aged less than two years, when protective antibodies against meningococcal serotypes have not developed. In patients with properdin deficiency, the median age at the time of meningococcal infection is much higher at approximately 14 years of age [74].

Although the risk of contracting meningococcal infection is significantly higher in individuals with properdin deficiency, not all will experience meningococcal infection during their lifetime [75]. Interestingly, a case of complete deficiency of factor D (FD) (autosomal recessive inheritance) was described in an adult with a history of *Neisseria meningitidis* infections following two episodes of disseminated gonococcal infection [76]. A familial case was reported in 2018, where members of the family had normal levels of factor D (FD) with decreased functionality due to a missense mutation [77]. In 2013, a 32-year-old woman with recurrent pneumococcal and meningococcal infection was diagnosed with factor B (FB) deficiency [78].

aHUS is a thrombotic microangiopathy characterized by hemolytic anemia, thrombocytopenia and renal failure, which occurs in the absence of its usual cause (infection with a shiga toxin-producing organism). aHUS is caused by primary factors such as mutations in complement genes and autoantibodies against complement regulatory proteins or secondary causes such as infection, drug toxicity or autoimmune disorders [79, 80]. Approximately 50–60% of cases of aHUS have an underlying genetic component that typically involves genes that regulate the AP:FH (20–30% of cases), factor I (FI) (5–10%), FB (1–4%), membrane cofactor protein (MCP)/CD46 (10–15%), thrombomodulin (3–5%) and C3 GoF (2–10%) [16, 70]. Approximately 20% of patients with aHUS have mutations in more than one gene [81], and patients with autoantibodies to regulatory proteins also comprise a significant subset. The majority of aHUS cases are sporadic and occur in the absence of prior family history. Furthermore, even in familial forms of aHUS, penetrance is incomplete [19].

Dysregulation of the alternative pathway appears to also play a significant role in the pathogenesis of AMD, which is the most common cause of vision loss in developed countries [82]. Various complement proteins and their activation products and regulators have been identified in the retinal deposits of patients with AMD. Pathology-driving polymorphisms in genes encoding for proteins of the complement system, particularly FH but also C3 and FI, have been associated with AMD [83].

## Lectin Pathway

### Lectin

The lectin pathway is focused on the recognition of repetitive carbohydrate patterns found on the surface of microbial pathogens. Lectin pattern recognition molecules (PRMs), which include MBL, ficolin-1, ficolin-2, ficolin-3, collectin-10 and collectin-11, activate the pathway in an analogous manner to antibodies in the classical pathway [11, 84–86]. MASPs, which act in a similar fashion to C1r and C1s, associate with MBL and activate C4 and C2 by proteolytic cleavage (Fig. 1) [14, 84].

Polymorphisms in the collectin and ficolin genes cause variable degrees of insufficiency and decreased serum concentrations [87]. Lectin pathway impairment due to insufficient production of any of these components is common and may be associated with no clear clinical phenotype, mild, incrementally or somewhat increased risk of infection [88] especially in young children and otherwise immunosuppressed individuals. There are likely to be other factors involved in defining severity, given that deficiencies in the lectin pathway or MBL alone did not decrease life span in large population-based studies [2]. Complete ficolin-3 (or H-ficolin) deficiency was initially associated with increased susceptibility to infections and necrotising enterocolitis. Due to its rarity, it is still unclear whether it is a life-threatening condition with variable penetrance or acts as a disease modifier [89]. Partial ficolin-2 (or L-ficolin) and ficolin-3 (or H-ficolin) insufficiencies are not well studied and are of uncertain clinical significance [90, 91].

### MBL

Among Caucasian populations, around 5–7% of people have inherited MBL deficiency (defined as less than 100 ng/mL) [13], although this threshold varies between countries/institutions) and does not affect overall mortality or increase susceptibility to community-acquired pneumonia [2]. However, MBL insufficiency is, in combination with other factors, observed in more severe forms of sepsis and fatal outcomes, irrespective of the causal microorganisms [92]. Low cord-blood MBL levels are weakly associated with respiratory symptoms during infancy [93]; however, MBL2 polymorphisms do not increase the risk of mortality following invasive meningococcal infection in children [94]. MBL insufficiency has been associated with increased frequency of pyogenic infections and/or heightened risk of sepsis in infants in some studies, as well as neutropenic patients undergoing chemotherapy and organ transplant recipients [88, 95, 96]. MBL2 polymorphisms may also be associated with increased susceptibility to recurrent infection with herpes simplex virus 2. MBL recognizes HSV, suggesting that MBL deficiency may be associated with frequently recurring HSV2 [97, 98]. A moderately-increased risk of acquisition or progression of other chronic viral diseases, chronic pulmonary aspergillus infections and severe malaria have also been previously associated with MBL insufficiency [88]. Conversely, low levels of MBL may confer resistance against mycobacteria [99]. MBL polymorphisms can also affect susceptibility to SLE and the risk of infection during treatment [100]; however, the role of MBL polymorphism in disease remains controversial.

### MASP1

MBL-associated serine protease 1 (MASP1), the most abundant protease of the lectin pathway, has a central role in pathway activation via MASP2. Several mouse models have shown links between MASP1 and coagulation, renal, gastrointestinal and myocardial ischemia/reperfusion-related pathology; however, as yet, there is no firm evidence for this type of pathology in humans [101, 102].

Malpuech, Michels and Mingarelli-Carnevale (3MC) syndrome is characterized by facial dysmorphia and other developmental defects such as cleft lip and palate, postnatal growth deficiency, cognitive impairment and hearing loss [13, 90, 91, 103]. It is caused by homozygous mutations in the MASP1 gene (3MC syndrome 1) or members of the collectin subfamily COLEC10 or COLEC11 (3MC syndrome 2) [103]. Excess or unusual infections and autoimmunity have not yet been described in this syndrome.

### MASP2

Severe MASP2 insufficiency was first observed in a single individual, together with anti-C1q autoantibodies, recurring pneumonia, pulmonary fibrosis, ulcerative colitis and erythema multiforme bullosum [104]. The frequency of MASP2 insufficiency caused by genetic polymorphisms is about 4% in Caucasians and up to 18% in some African populations [105]. However, most MASP2-insufficient individuals are asymptomatic. MASP2 insufficiency has also been associated with increased risk of fever and neutropenia in pediatric patients undergoing chemotherapy [106]. A meta-analysis showed that a common polymorphism that affects serum levels of MASP2 was not associated with the development of infectious disease [107] Additionally, MASP2 deficiency may be associated with prematurity and low birthweight but not with perinatal infections [91].

## Deficiencies of Complement Regulation

The complement system has several levels of regulation at the initiation, amplification (formation of convertases) and membrane attack phases, thereby preventing inadvertent tissue damage [15, 108]. Deficiency of complement inhibitors leads to dysregulation either in the fluid phase or on cell surfaces and consequent recurrent infections (mostly bacterial), inflammatory disorders and presentations with a broader clinical phenotype. These include angioedema (C1 inhibitor [C1-INH] deficiency), kidney and eye diseases (FH, FI or CD46 deficiency), protein-losing enteropathy (CD55 deficiency) and paroxysmal nocturnal hemoglobinuria (PNH) (CD55 + CD59 deficiency) [1, 108, 109]. It is important to note that, in contrast to complete deficiencies of complement regulatory proteins that result in consumption of multiple components in a pathway, haploinsufficiency can cause excessive local inflammation occurring at sites of tissue injury or debris accumulation [110]. For example, haploinsufficiency of FH predisposes to aHUS and AMD [111–116], while homozygous FH deficiency results in alternative pathway activation, cleavage and consumption of C3 and FB and increased susceptibility to pyogenic infections. Heterozygous deficiency of FI is also associated with both aHUS and AMD [112, 113].

Haploinsufficiency (50% of normal functional capacity) of complement regulators creates a “hyperinflammatory” phenotype driven by the feedback loop of the alternative pathway (Fig. 1), sometimes described as the complement inflammasome. Loss of function in a plasma or membrane inhibitor of the alternative pathway results in excessive activation of complement on the endothelium of the kidney in aHUS and, in AMD, the accumulation of retinal debris within drusen and complement-mediated inflammation.

In aHUS, the disease commonly presents due to haploinsufficiency of one of three complement regulators FH, MCP or FI. Several mechanisms have been described, the loss of functional capacity due to haploinsufficiency itself and autoantibodies, which block regulatory proteins (autoantibodies against FH occur in 10% of adult aHUS cases). For reasons that are not fully understood, autoantibodies usually develop against a background of deletion of complement FH related protein (CFHR) 1 and CFHR3 (Table 1). In addition, the C3 and C5 convertases of the AP can be stabilized (with a prolongation of half-life) due to mutations in C3 or FB, most occurring in the binding site. Lastly, polymorphisms in non-coding regions of complement regulators may predispose to disease secondary to effects on expression; however, further studies are needed to demonstrate this definitively [110].

Deficiency of C1-INH results in episodic angioedema without urticaria that is inherited (hereditary angioedema (C1-INH-HAE) or acquired (C1-INH-AAE) [117, 118]. In addition to its role as an inhibitor of C1r and C1s of the classical pathway and MASP1 and MASP2 of the lectin pathway, C1-INH is the major inhibitor of factor XIIa and kallikrein. The lack of inhibition of these enzymes results in excessive bradykinin generation, which, in turn, increases vascular permeability, leading to angioedema [118, 119]. However, a number of patients suffering from angioedema without wheals present with normal C1-INH levels. Mutations in factor XII (FXII), plasminogen (PLG), angiopoietin-1(ANGPT1) and kininogen-1 (KNG1) have been found in this newly-defined group of primary angioedema patients with normal C1-INH (nlC1-INH) [120]; however, a significant proportion does not have a defined molecular explanation as of yet (HAE-unknown) [121, 122].

FH and FI are key regulators of the AP. Deficiency of either of these regulators is associated with recurrent infections and results in uncontrolled activation of the AP with subsequent secondary C3 deficiency and a reduction in circulating FH levels (when FI is deficient) [123, 124]. Distinct clinical manifestations have been associated with mutations in several complement components. Approximately 50% of patients with aHUS have genetic mutations of FH, FI, C3, FB and/or MCP and deletion of complement FH-related proteins 1 and 3 (CFHR1/CFHR3) [109]. Thrombomodulin also has a regulatory role and binds to FH and C3b thereby inhibiting complement activation [125]. Interestingly, mutations in FH, MCP and FI have also been reported in C3 glomerulopathy (formerly known as membranoproliferative glomerulonephritis, MPGN) [109], as well as pre-eclampsia [126] and hemolysis, elevated liver enzyme levels and low platelet levels (HELLP) syndrome [127]. Partial FI deficiency has also been previously associated with clinical manifestations including recurrent tonsillitis, urinary infections, otitis, pyelonephritis, severe meningitis and sepsis [128].

Decay accelerating factor (CD55) is a membrane-bound regulator that dissociates both classical and alternative C3 convertases. CD59 is the key membrane regulator of the terminal pathway that prevents insertion of the MAC into host tissue. Somatic mutations in the phosphatidylinositol glycan class A (PIG-A) gene coding for the anchoring structure of both inhibitors leads to PNH [129]. Isolated congenital CD55 deficiency is rare but has been observed in patients suffering from severe early-onset protein-losing enteropathy [130], whereas severe Guillain-Barré-like neurological symptoms with hemolysis are the hallmark of isolated CD59 deficiency [131].

## Management and Treatment of Complement Deficiencies

Recognition of the following warning signs may help clinicians in the diagnosis of complement deficiencies [1]:

- Meningococcal meningitis at > 5 years of age
- Recurrent systemic bacterial infections with encapsulated organisms (particularly *Streptococcus pneumoniae* and more rarely gonococcal disease)
- Autoimmune diseases (particularly SLE)
- Angioedema without urticaria
- Inflammatory disorders involving the kidney or eyes
- Unusual infections, e.g., epiglottitis despite vaccination against *Haemophilus influenzae* type b (HIB)
- Severe infection with encapsulated bateria

Recurrent infections with encapsulated bacteria, especially pneumococcus as well as neisserial infection, should alert clinicians to early or late complement component deficiencies, respectively [132], while renal disorders such as aHUS and MPGN may suggest dysregulation of the AP [7]. Autoimmune manifestations are generally associated with early classical pathway component deficiencies, while angioedema without urticaria should alert the clinician to investigate C1-INH deficiency [49]. Immunologists should help raise awareness of these rare disorders among generalists and specialists, and diagnosed patients should receive education about how to recognize complications and when to seek medical attention.

It is important to note that similar management principles for the prevention or treatment of infection may apply in settings where complement deficiency is secondary to a targeted complement inhibitor such as eculizumab (anti-C5 mAb) or consumption of C3 that occurs in the presence of C3 nephritic factor.

Except for HAE, replacement therapy has never reached routine clinical practice, in part due to rapid metabolism of complement proteins. Approaches to the management of complement deficiencies depend on the specific disease involved.

### Ongoing Management and Family Assessment

Obtaining a detailed family history is useful for the diagnosis and management of complement deficiencies associated with immunodeficiency. Most heritable complement deficiencies are autosomal recessive, and carriers are asymptomatic. Exceptions include X-linked properdin deficiency and autosomal dominant FB, C1-INH and MCP/CD46 deficiencies and with haploinsufficiency of complement regulatory proteins (FH, FI) [1]. Testing of siblings and other potentially affected family members is recommended as relatives benefit from the same preventive care as patients. There are several important points to consider regarding education of the patient and family to ensure optimal care and management:

- How often patients should be followed up
  - Annual follow up is recommended following diagnosis of a complement deficiency in order to provide education, up-to-date advice on appropriate vaccination, antibiotics as needed, advice for emergencies and family studies as needed
- The potential use of MedicAlert® or similar bracelets to facilitate early recognition of the underlying disorder if patients become unwell
  - The MedicAlert® website has more information on how to join and ensure fast, accurate treatment in an emergency (https://www.medicalert.org/)
- Guidance for patients regarding pregnancy, travel and surgical procedures etc.
  - In pregnancy, complement deficiency can increase the risk of preeclampsia [133]
  - During pregnancy, patients should have up-to-date vaccinations (if not already vaccinated) and an emergency plan in place in the event of infection. Patients should be informed about inheritance of complement deficiencies and any subsequent testing that may be needed
  - It is recommended that patients talk to their immunology health team at least three months before they travel. They will be able to advise the patient which vaccinations they might need and what medication to take with them [134, 135]
  - It is recommended that patients with complement deficiency be closely monitored following surgery with early recourse to assessment and antibiotic treatment. In a study of 538 splenectomised patients, 38 patients developed bacteremia during the first month after surgery [42, 136]
  - In addition, pre-procedural prophylactic administration of C1-INH concentrate is recommended for HAE patients undergoing surgical and dental procedures [49]
- Estrogen may exacerbate angioedema attacks in HAE and patients should be advised to avoid combined contraceptives and hormonal replacement therapy [49]
- Agree an emergency plan in the event of infection and inform the other clinical teams involved in primary and secondary care. This plan may involve early recourse to medical attention and an emergency supply of antibiotics
  - Complement-deficient patients are at increased risk of infection; therefore, it is of paramount importance to explain to patients their predisposition to infection and the importance of preventative immunisations and prompt recourse to antibiotic therapy in line with their emergency plan

### Vaccinations

In patients with complement deficiency, the same vaccines are recommended as in healthy individuals, with particular emphasis on conjugated vaccines against pneumococcus, *Haemophilus influenzae* and *Neisseria meningitidis* [137, 138]. Unconjugated polysaccharide vaccines do not elicit a memory response and are not immunogenic in children under the age of 2 years [42]. Inducing and maintaining humoral immunity through vaccination enhances host defenses where complement is lacking [139]. The tetravalent conjugate vaccine against the serogroups A, C, Y and W of *Neisseria meningitidis* is strongly recommended for patients with complement deficiencies [1, 18, 42, 140], together with the meningococcal B vaccine [140]. Contacts should also be vaccinated [138, 141]. No vaccines are contra-indicated in patients with complement deficiencies, meaning that live vaccines can be administered. The efficacy of vaccines in patients with complement deficiencies has not been evaluated in large cohort studies; however, vaccinations are recommended by the Advisory Committee on Immunization Practices [138, 142]. Guidelines change frequently with accumulating experience and are best accessed in real time. C3 is the major opsonin in the complement pathway; deficiency of which can result in an increased susceptibility to invasive pneumococcal infections and recurrent pyogenic infections [143]. In addition to the vaccination advice above, there is a need to identify low pneumococcal antibody levels in patients with C3 deficiency or rare C3 deficiency syndromes, e.g., C3 nephritic factor and FH deficiency. This facilitiates appropriate, individualized booster vaccinations with conjugate or polysaccharide pneumococcal vaccines.

We recommend monitoring vaccine responses where possible and administering boosters depending on the durability of protective antibody levels.

For patients with HAE who may require blood products as part of their therapy, hepatitis B vaccination is recommended.

### Antibiotics

The use of antibiotic prophylaxis in complement deficiencies is aimed at protecting against infection by encapsulated organisms, and it is best reserved for patients exhibiting recurrent infections despite appropriate vaccination [18, 42]. This approach was supported in a prospective study of patients with homozygous C6 deficiency and recurrent infections living in an area with endemic group B meningitis, where the use of monthly benzathine penicillin protected against further neisserial infections [42]. For patients not considered to require a prophylactic regimen, it is advisable to ensure access to emergency antibiotics and prompt medical review as part of their emergency plan for encapsulated bacterial infections. Importantly, patients with C3, FH or FI deficiency who have been vaccinated against meningococcal disease may still present with infections.

In summary, the decision to offer antibiotic prophylaxis (e.g., penicillin- or macrolide-based) over emergency antibiotics should be individualized based on risk stratification. Patients who have high exposure to bacteria (e.g., those living in endemic areas or working in high-risk professions such as nursery care) and have recurrent infections may be selected for prophylaxis. The benefits of prophylaxis should be balanced against the risks, such as potential development of resistance to the antibiotics used.

### Complement Inhibitors

Eculizumab is a recombinant humanized monoclonal antibody with targeted activity against human C5, which inhibits the cleavage of C5 and subsequent formation of the MAC [7, 12, 144]. Eculizumab is very effective in the treatment of PNH and of aHUS, preventing progression to end-stage renal disease [10, 19, 80]. However, a potential major side effect is recurrent meningococcal infections, and patients must therefore receive the meningococcal vaccines prior to initiating therapy and have access to antibiotic prophylaxis [7]. The cost of therapy is estimated at $600,000 (approximately €529,000) per year but must be considered against the costs otherwise incurred including plasma exchange, hospitalisations, end-stage renal disease and impaired quality of life [7]. Monitoring of eculizumab includes functional analysis of the classical complement pathway, alternative pathway and complement activation products including C3d and sC5b–9/TCC [30]. Only recently, ravulizumab, another humanized monoclonal anti-C5 antibody, has been approved for the treatment of PNH by the US FDA and is currently under regulatory review in both the European Union and Japan [145]. Phase 3 development of intravenous ravulizumab for the treatment of aHUS is underway.

Treatment for HAE has improved with the development of C1-INH replacement as well as other agents designed to overcome the effects of C1-INH deficiency [12, 144]. C1-INH therapy is currently available in several formulations including intravenous (IV) C1-INH replacement administered prophylactically (Cinryze®), IV C1-INH that is used to treat acute episodes of facial, abdominal and laryngeal attacks (Berinert®), IV C1-INH replacement that is used to treat acute episodes in adults and adolescents (Ruconest®) [146] and subcutaneous C1- INH that is used for routine prophylaxis in adolescent and adult patients (Haegarda®) [147]. Alternatives to C1-INH for acute therapy include Icatibant (Firazyr®, a bradykinin B2 receptor antagonist) and Ecallantide (Kalbitor®, available in the US), which are both used for the treatment of HAE [146]. Lanadelumab (Takhzyro®) is a monoclonal antibody against kallikrein that received approvals for the prophylactic treatment of HAE in 2018 [148] and approved by the National Institute of Clinical Excellence (NICE) in 2019 [149]. Tranexamic acid, androgens, fresh frozen plasma and solvent detergent-treated plasma have been historically used in the treatment of HAE; however, for reasons such as limited efficacy and risks of adverse events, these agents are no longer recommended except as a last resort if no better choice is available [49]. The recent WAO/EAACI guideline includes detailed information regarding the recommended treatments for patients with HAE [49].

### Gene Therapy

Gene therapy has been evaluated in preclinical models of HAE. A one-off intravenous administration of an adeno-associated virus vector expressing the normal genetic sequence of human C1-INH was effective at maintaining a normal level of circulating C1-INH in mice and is hypothesized to provide long- term protection from angioedema attacks in patients with HAE [150].

### Hematopoietic Stem Cell Transplantation (HSCT)

C1q is produced by monocytes in healthy individuals; therefore, HSCT serves as a potentially curative intervention for C1q deficiency. Owing to the scarcity of data, there are no evidence-based recommendations on how best to use HSCT in patients with complement deficiencies as further studies are required. However, several cases have been reported in which HSCT has resulted in restoration of functional C1q and resolution of SLE symptoms [151, 152]. There have also been some reports of favorable responses to HSCT in HAE where hematopoietic production of C1-INH appeared sufficient to prevent attacks [153, 154] and for alloSCT in acquired angioedema [155]. As an alternative to HSCT, combined liver and kidney transplantation has the potential to correct aHUS if the proteins encoded by the deficient genes are predominantly synthesized in the liver, such as FH or FI [156]. Importantly, management of C1q deficiency should take into account individual patient requirements as not all interventions are likely to be successful for all patients [157].

## Conclusion

Knowledge regarding the complexity and clinical implications of defects in the complement system as well as the effects of novel therapeutic agents targeting complement continues to grow. Most complement deficiencies, with certain exceptions, are rare, and the clinical presentations are protean (e.g., infections, angioedema and renal, connective tissue, ocular, neurological, gastrointestinal and hematological diseases). Challenges are presented by the small numbers of patients with these deficiencies, but advances are being made in relation to the availability of diagnostic testing, standardization of complement testing and next generation sequencing. There is also an active pipeline of therapies in development for the treatment of disorders due to complement deficiencies as well as complement-mediated inflammation. Complement deficiencies represent an exciting field of medicine that is changing rapidly. This guideline reflects the evidence available in 2019, and it is anticipated that updates will be required every 2 to 3 years to reflect the developing evidence and changing practice.
